# Nonsurgical Transurethral Radiofrequency Collagen Denaturation: Results at Three Years after Treatment

**DOI:** 10.1155/2011/872057

**Published:** 2011-12-05

**Authors:** Denise M. Elser, Gretchen K. Mitchell, John R. Miklos, Kevin G. Nickell, Kevin Cline, Harvey Winkler, W. Glen Wells

**Affiliations:** ^1^Illinois Urogynecology, LTD., 5716 West 95th Street, Oak Lawn, IL 60453-2345, USA; ^2^Atlanta Urogynecology Associates, Alpharetta, GA 30005, USA; ^3^SOGA, Houston, TX 77004, USA; ^4^Regional Urology, LLC, Shreveport, LA 71106, USA; ^5^North Shore Women's Health, Great Neck, NY 11021, USA; ^6^Alabama Research Center, LLC, Birmingham, AL 35209, USA

## Abstract

*Objective*. To assess treatment efficacy and quality of life in women with stress urinary incontinence 3 years after treatment with nonsurgical transurethral radiofrequency collagen denaturation. *Methods*. This prospective study included 139 women with stress urinary incontinence due to bladder outlet hypermobility. Radiofrequency collagen denaturation was performed using local anesthesia in an office setting. Assessments included incontinence quality of life (I-QOL) and urogenital distress inventory (UDI-6) instruments. *Results*. In total, 139 women were enrolled and 136 women were treated (mean age, 47 years). At 36 months, intent-to-treat analysis (*n* = 139) revealed significant improvements in quality of life. Mean I-QOL score improved 17 points from baseline (*P* = .0004), while mean UDI-6 score improved (decreased) 19 points (*P* = .0005). *Conclusions*. Transurethral collagen denaturation is a low-risk, office-based procedure that results in durable quality-of-life improvements in a significant proportion of women for as long as 3 years.

## 1. Introduction

The management of female SUI [1, 2] continues to evolve with improvements in both our understanding of the continent mechanism [3] and with improvements in the technologies and methods used to treat incontinence. Of particular note, the introduction of synthetic slings in the mid 1990s and the widespread use of urodynamics have contributed to improved management of patients who experience SUI. However, although SUI remains a pervasive problem in the female population with a prevalence of 25% to 30% [4], fewer than 5% of US women diagnosed with SUI opt for surgical intervention. 

Despite its prevalence and the associated distress, embarrassment, and diminished quality of life (QOL), many women who experience symptoms of SUI delay or do not seek medical treatment. A recent survey found that only 15% of women aged 40 years or older with SUI sought medical treatment for their symptoms [5]. Although personal reasons for not seeking treatment vary among individual women, they include fear of the risks and life disruptions associated with current surgical options [6], and an interest in less-invasive treatments offering symptom improvement versus cure is growing [7]. 

In keeping with this trend, and reflecting the growing interest by physicians in procedures that can be performed in-office, this study reports on the 36-month durability of an incision-free treatment for SUI primarily intended for symptom relief. This treatment, which is typically performed in an office setting [8, 9] using local anesthesia (Renessa; Novasys Medical, Inc, Newark, Calif, USA) received FDA clearance in 2005 following a randomized, sham-controlled study by Appell et al. [10] that demonstrated both safety and efficacy at 12-month after treatment. The introduction of this technology has meant that patients now have a convenient one-time, in-office, nonsurgical treatment option for the management of their SUI. 

Expectations about outcomes (expressed in terms relevant to women's QOL) influence the choice of treatment once she does seek treatment, since each therapeutic option is associated with varying degrees of invasiveness, effectiveness, and expected impact on QOL both during recovery and afterward. As such, most studies of SUI therapies now include both objective and subjective measures, such as validated QOL questionnaires, which may also be used in clinical practice [11]. 

Noninvasive conservative (behavioral) options or physiotherapy are typically selected as first-line therapies. Although such interventions can be effective and can substantially improve a patient's QOL [12–15], the onset of benefits may be prolonged, and successful outcomes depend on the patient's understanding, training, motivation, and persistence [13, 15, 16]. More passive treatment using devices such as vaginal pessaries and cones/weights may also improve SUI symptoms [17, 18]. 

Certain drugs, such as estrogens, *α*-adrenergic receptor agonists, *β*-adrenergic receptor antagonists, tricyclic antidepressants, anticholinergics, and a serotonin-norepinephrine reuptake inhibitor are prescribed off label by some US physicians, but they are not consistently effective and none is currently approved by the US Food and Drug Administration (FDA) for the treatment of SUI [6]. Additionally, as with any drug, there may be a risk for long-term side effects. In a study by Robinson et al., only 14% of women surveyed were willing to accept long-term drug therapy [7]. 

Periurethral injection of bulking agents is a nonsurgical treatment option that may be used in women with SUI due to intrinsic sphincter deficiency (ISD). While most studies of bulking agents report good initial efficacy and improvements in QOL, the durability of effect can decrease over time [12, 19] and may require repeated injections [12, 19, 20], The newer synthetic agents may provide better durability than collagen-based agents [21, 22]. Safety appears to be generally good, but concerns exist about the long-term effects of repeated injections [12]. For example, fibrosis and/or denervation of the urethral sphincter from repeated injections could interfere with the ability to perform subsequent surgery for recurrent incontinence [12]. Additionally, some serious adverse events, such as particle migration and mass formation, have been reported with some synthetic bulking agents [23–26]. 

Surgical procedures are popular treatment options that can be safe and effective, but none is without risks or side effects, and each requires a recovery period of several weeks before the patient can return to normal daily activities. Furthermore, voiding difficulties, detrusor overactivity, and other adverse events are well-described complications following SUI surgery [27], and some synthetic sling materials have been associated with erosion into the vagina or urinary tract [12]. 

Effective results for the transurethral collagen denaturation treatment have been previously reported [10, 28, 29], thus the present study was prospectively designed to further assess the durability of results at 36-month following the procedure performed in the typical office-practice setting. This paper describes a prospective analysis of treatment efficacy and impact on quality of life, primarily focused on results at 36 months; however, results from previous time points are also reported.

## 2. Materials and Methods

This 36-month, open-label, single-arm clinical trial was conducted at multiple US physicians' offices to evaluate the long-term safety and efficacy of transurethral radiofrequency collagen denaturation. Women who were clinically diagnosed with SUI secondary to bladder outlet hypermobility for 12 months or longer were eligible for inclusion. Clinical evaluation and diagnosis were made by each investigator based on diary data, quality of life questionnaires, in-office stress pad-weight test, patient history, and physical examination. Urodynamic studies were not required but were employed at the investigators' discretion if determined to be necessary to make or confirm a clinical diagnosis. Participants, aged 18 years or older at baseline, had failed traditional nonsurgical therapy and were not candidates for surgical treatment (including women who wished to avoid surgery) and had the ability to complete all study requirements. Patients were determined to have failed traditional conservative therapy if they indicated that they tried and failed pelvic muscle exercises, pharmacotherapy, or biofeedback. Exclusion criteria included pregnancy, prior SUI surgery or bulking agent injection, leak point pressure less than 60 cm H_2_O, postvoid residual bladder volume greater than 50 mL, stage IV pelvic organ prolapse, and/or a diagnosis of detrusor overactivity or primarily urge incontinence. 

As SUI is a clinical diagnosis, this study was designed to be applicable to a general SUI population and to current office-based clinical practice, thus clinical assessment was used. 

The protocol was approved by Western IRB (Protocol number 20052168) in December 2005 and written informed consent was provided by all study participants. 

The majority of transurethral collagen denaturation procedures were performed in the study physician's office using local anesthesia. Patients received a prophylactic oral antibiotic (levofloxacin 250 mg or ciprofloxacin 500 mg, or appropriate alternative) 3 to 4 hours before treatment and an oral sedative (diazepam 5 to 10 mg or similar benzodiazepine) 30 to 90 minutes beforehand. Patients were placed in the lithotomy position, with a return “grounding” pad applied to the skin, and given a periurethral lidocaine block (10 cc of 2% lidocaine with epinephrine 1 : 100000 or 1 : 200000). The radiofrequency probe was then inserted transurethrally until the tip was positioned within the bladder lumen, and the balloon at the probe tip was insufflated. From the probe shaft, four 23-gauge nickel-titanium needle electrodes were deployed. Using deployment markings on the probe to guide rotation, and the balloon and needles to position the device, radiofrequency energy was delivered through the needles for nine 60-second cycles as sterile, room-temperature water irrigated the urethral mucosa. During each cycle, the submucosa surrounding the 4 needle tips was heated to 65°C, producing local collagen denaturation at 36 sites circumferentially around the bladder neck and proximal urethra without tissue necrosis [10, 30]. Upon completion of the final cycle, the needles were retracted into the shaft, the balloon was deflated, and the probe was removed. The patient was asked to void following the procedure, which was typically completed in about 30 minutes. Most patients left the physician's office within 1 hour after treatment and returned to normal activities the same day or the next. 

Reflecting the study's goal of measuring durability of the improvement in symptom relief using a metric relevant and important to patients, the primary efficacy end point was that at least 60% of patients would experience a 50% or greater reduction from baseline in stress incontinence episodes at 12 months. As previously reported, this was achieved [28]. Secondary measures included changes in incontinence impact on QOL assessed using the validated incontinence qualify of life (I-QOL) instrument [31] and on the urogenital distress inventory (UDI-6) scale. Each of these assessments was conducted at baseline and at 3, 6, 12, 18, 24, and 36 months. If patients did not return to the physician's office for the 36-month visit, they were contacted, per protocol, and asked to complete the I-QOL and UDI-6 instruments via telephone. 

Of particular interest are results obtained using intent-to-treat (ITT) analysis, wherein data from all 139 patients initially enrolled are included. For such analysis, the missing value strategies employed were as follows: (1) for intermittent missing data, the mean change from the nearest available adjacent time point prior to the time point with missing data from among those patients with data present for both time points was calculated; this same change was then applied to the missing data point to obtain an imputed data value; (2) for truncated missing data, wherein a patient was not available beyond a certain time before study completion, multiple imputations (MIs) were employed. A logistic regression model was fit to estimate the probability of a patient having a missing data point (propensity score). Baseline characteristics in the model included age, weight, baseline leaks associated with activity, pelvic prolapse stage, baseline I-QOL score, and baseline UDI-6 score. Additionally, values for the outcome of interest at all available follow-up time points were included in the model. Patients who were similar with respect to their probability of having a missing data point were grouped together into 5 strata using quintiles of the propensity score. Since the LR model includes both baseline and available data, patients were grouped on both their baseline factors and on their similarity in trajectory over time for the outcome variable in question. Next the missing data point was generated using an imputed value assigned by randomly choosing a value from among those in the same stratum from patients with nonmissing observations. The procedure was repeated 20 times on the entire data set, resulting in 20 different complete data sets. Missing-value estimates and the corresponding standard errors for each outcome variable (e.g., mean change from baseline to 36 months) were then calculated from each of the 20 data sets by combining the between and within imputation variance. So the resulting estimate incorporates both the between subject variability and the variability associated with the imputation method to provide an overall unbiased missing-value and standard error estimate that accounts for missing data uncertainty and includes data from all 139 patients.

Mean changes from baseline at 36 months for I-QOL, UDI-6, pad weight, leaks/day per bladder diaries, and daily leaks due to activity obtained from case report forms were assessed using a paired student's *t* test. Over the extended follow-up period, the percentages of patients were described with (1) at least a 10-point improvement in I-QOL, (2) any improvement in I-QOL, (3) at least a 50% reduction in pad weight, (4) “dry” pads using the ICS convention for this definition, and (5) at least a 50% reduction in leaks per day using diary analysis. SAS version 9.1 (SAS; Cary, NC, USA) was used to complete all analyses.

The incidence of device-related and of serious adverse events were the primary safety end points.

## 3. Results

**Figure 1:**
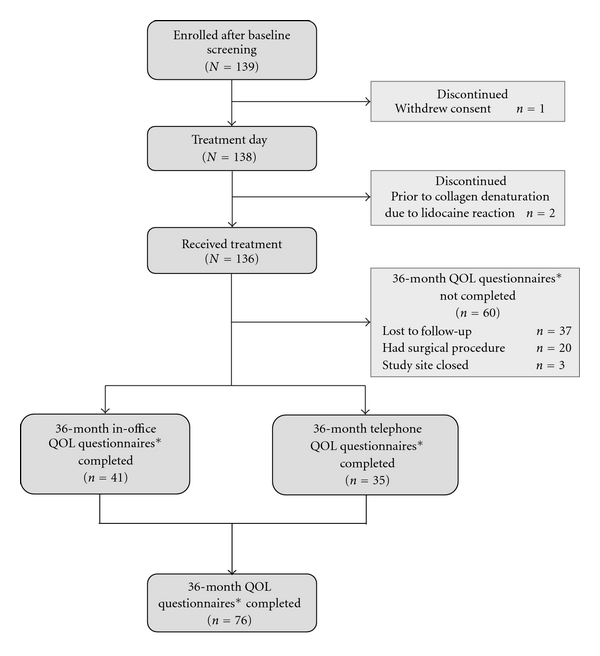
Patient disposition. *Two validated questionnaires, the incontinence quality of life and the urogenital distress inventory instruments, were completed at 36 months.

The study enrolled 139 women at 13 centers.  Table 1 reports baseline demographics and characteristics. Prior to treatment, 1 patient withdrew consent and the remaining women continued into the treatment phase. Two women were discontinued prior to undergoing transurethral collagen denaturation because of lidocaine reactions. The remaining 136 women underwent treatment (Figure 1). All 139 enrolled women are included in the ITT analysis.

As shown in Figure 1, 20 patients were known by investigators to have undergone subsequent surgery, and 3 patients were lost to follow up when their study site closed. Of the remaining 113 patients, 41 completed their scheduled in-office evaluations at 36 months after treatment. Investigators then attempted to contact the 72 remaining patients to ask them to complete both I-QOL and UDI-6 quality-of-life measures via telephone. Each pair of QOL surveys took approximately 10 minutes to complete via telephone. Previous studies have validated the concordance of telephone versus in-person QOL survey results [32, 33], including results with the pelvic floor distress inventory, which is based upon the UDI questionnaire [11]. 

Of the 72 patients contacted, 35 women responded and completed the I-QOL and UDI-6 questionnaires. Thirty-seven women could not be located and were thus considered lost to follow up.

The baseline characteristics of women who underwent treatment but failed to provide 36-month data did not differ from those who continued in the trial. 

### 3.1. Efficacy

Reduction in leaks from baseline was assessed using a patient bladder diary (Tables 2 and 3). In the ITT population, the estimated percentage of patients with a 50% or greater reduction from baseline in leaks per day equaled 60%; the mean baseline of 3.2 leaks per day declined to an estimated 1.5 leaks per day by month 36 (*P* = .001), continuing the positive trend first reported at 12 months [28].

I-QOL results are shown in Tables 4, 5, and 6. For the ITT population at 36 months, improvement from baseline averaged 16.5 points (*P* = .0004), with an estimated 60% of patients achieving at least a 10-point improvement in I-QOL scores and 71% achieving some improvement. A 10-point improvement in the I-QOL score has been shown to be clinically meaningful and is associated with a 25% reduction in pad weight and a 25% reduction in incontinence episodes [34]. 

The ITT I-QOL results for estimated percentages of patients achieving at least a 10-point improvement were stratified by severe, moderate, and mild at baseline (0–30, 31–60, 61–90, resp.; Table 6). Among patients with moderate/severe SUI baseline values, 74% had at least a 10-point improvement in I-QOL scores. 

With regard to UDI-6 results, the mean score at baseline of 52.7 declined by 33.3 in the ITT population at 36 months (*P* = .0005, Table 7).

Results for pad weight are described in Tables 8, 9, and 10. For the ITT population, mean baseline pad weight decreased from 35.5 g at baseline to 23.0 g (*P* = 0.18), with an estimated 57% of patients achieving at least a 50% reduction in pad weight. Defining a “dry” pad as one weighing less than 1 g, 35% of ITT patients were considered dry at 36 months. 

To explore the impact of different assumptions about withdrawals and missing data, a sensitivity analysis was performed for the key outcome variable success rates (Table 11). This analysis used a multiple imputation method wherein known surgery patients are always considered failures, with each patient's final presurgery data carried forward. Multiple imputation was then applied to all remaining truncated data alternately treating the 37 patients lost to follow up as successes and as failures. As expected, for the outcomes with the most missing data, the range of possible outcomes was wide, reflecting the limits of the two most extreme scenarios. Since neither of these two extremes is likely to happen, the multiple imputation method provides an appropriate estimate of what is likely to be the correct value, located between these extremes, reflecting both the observed data and the variability of the estimate. 

### 3.2. Safety

Transurethral collagen denaturation was shown to be safe and well tolerated for at least 36 months after treatment. Immediately following the procedure, about one-third of patients were able to return to normal daily activities within 1 day of treatment (most within a few hours), while 125 (91.9%) patients returned to normal activities within 2 days. At day 3, the most common adverse events included dysuria (*n* = 7; 5.2%), urinary retention (*n* = 6; 4.4%), postprocedure pain (*n* = 4; 2.9%), and urinary tract infection (*n* = 4; 2.9%). These events were transient and resolved quickly. One patient required an indwelling catheter for 24 hours. Average pain score reported on a 10-point visual analog scale (VAS) was 1.6. At 12 months, one patient reported an increase in leaking, which the investigator deemed as probably related to treatment. Symptoms of urgency or urge incontinence were reported by 8 patients; in 6 patients, these symptoms resolved by 12 months, and in 1, by 25 months; symptoms remained in 1 patient at 36 months.

At 18 months, 1 patient experienced a myocardial infarction that was deemed by the investigator to be not related to the procedure. At the 24-month visit, one patient reported a dry cough that was deemed not related to the procedure, and another patient reported a recurrence of SUI. No new treatment-related adverse events were reported between 18 and 36 months.

## 4. Discussion

Results from this 36-month study of nonsurgical transurethral collagen denaturation in women with SUI due to bladder outlet hypermobility demonstrated the efficacy, durability, and safety of this procedure. 

Validity studies have shown that both the I-QOL and the UDI-6 questionnaires can be used successfully in both daily practice and in clinical research, and that these instruments reliably assess the impact of symptom severity on patient quality-of-life [34–37]. One study showed that 2.5- and 6.3-point differences in I-QOL scores indicate significant improvement in clinical status, as demonstrated by a reduction in the frequency and severity of incontinence episodes and by improvement in the patient's global perception of her condition [34]. A 10-point increase generally indicates a patient's perception of being “much better” and is associated with a 25% or more reduction in both the frequency and severity of incontinence episodes. Studies of the UDI-6 show that results correlate well with urodynamic diagnoses, while additional studies have reported similar results in correlating QOL instruments with quantitative measures [35, 38, 39]. 

In the present study, the significantly reduced number of leaks at 36 months versus baseline demonstrated durable improvement at 36 months after treatment. Patient QOL scores also improved significantly from baseline, as shown by favorable results in both the IQOL and UDI-6 scores, indicating a significant improvement in SUI symptoms. There was an overall low incidence of adverse events through 36 months, with no serious procedure-related adverse events noted at any time point. Patients reported a rapid return to normal daily activities following the procedure. 

Stratifying the baseline incontinence severity by I-QOL and using a 10-point improvement (on a 100-point scale) as an efficacy criteria, both an earlier 12-month, randomized, sham-controlled trial [10] and the current 36-month study demonstrated the effectiveness of this treatment in patients with moderate to severe incontinence (baseline I-QOL score, <60). In the earlier study, 74% of patients achieved a 10-point IQOL improvement compared with 50% of patients who underwent the matching sham treatment. At 36 months in the present trial, a 10-point or greater I-QOL improvement from baseline was again achieved by 74% of all patients with this degree of baseline severity.

A limitation of this study includes the dropout rate with regard to patients completing all of the in-office assessments. Unfortunately, high dropout rates are very common in trials of incontinence treatments. Recent publications have reported retaining only approximately 50% of patients at 2 years and 35% at 5 years (for primary outcomes) [40–44]. These high dropout rates are possibly related to patients' unwillingness to subject themselves to the inconvenience and unpleasantness of evaluations such as UDS and pad weight tests or the nuisance of recording bladder diaries. Anticipating patient dropouts, the investigators in this study included the option of administering questionnaires by phone to obtain more complete data. Results are also reported on an ITT basis, including all enrolled patients, following application of a missing-value imputation technique appropriate for long-term studies where the improvement rate changes over time. Sensitivity analyses were also performed to explore differences in outcomes based on best- and worst-case assumptions regarding the outcomes of patients for whom no data was available. 

Data from patients who were known to have undergone subsequent surgery showed that despite being considered “treatment failures,” half of these patients (10/20) had reported some level of improvement following transurethral collagen denaturation. Additionally, if a patient's intention was to prolong the need for surgery and not to avoid surgery altogether, the duration of time between the procedure and surgery could have been viewed as a successful outcome.

Overall, results of the present study support and extend those of previous studies of transurethral collagen denaturation. It is a minimally invasive, safe procedure that can be performed under local anesthesia in a physician's office, with negligible to minimal recovery time. Results from this study also indicate that transurethral collagen denaturation does not preclude subsequent surgery for SUI if a patient so desires. These traits make this procedure an attractive nonsurgical treatment option for women with SUI who have failed conventional conservative therapies but who cannot undergo or wish to avoid surgery. 

## 5. Conclusions

Results from this 36-month study show that woman who underwent nonsurgical transurethral collagen denaturation experienced significant and durable improvements in QOL. The long-term durability of this minimally invasive procedure in women with SUI due to bladder outlet hypermobility indicates that transurethral collagen denaturation may be a beneficial intervention for women with this condition who wish to avoid or postpone surgery. The results also confirm that the treatment has a good safety profile, with no serious adverse events reported at any time during this or previous trials.

## Figures and Tables

**Table 1:** Baseline characteristics of the study population.

Characteristic	All enrolled patients
	(*N* = 139)
Median age, y (range)	47 (26–87)
Median weight, lb (range)	160 (110–280)
Mean total I-QOL score (SE)	51.2 (1.7)
Mean UDI-6 score (SE)	52.7 (1.4)
Median leaks caused by activity (range)	
Daily	2.9 (0–11)
Weekly	20 (0–80)
Pelvic organ prolapse stage, *n* (%)	
Stage 0	45 (33%)
Stage 1	63 (45%)
Stage 2	29 (21%)
Stage 3	1 (0.7%)
Unknown	1 (0.7%)

I-QOL: Incontinence Quality of Life; UDI: Urogenital Distress Inventory.

**Table 2:** Stress Leaks per day per diary.

Visit	Directly Evaluable		Intent-to-Treat	
	*N*	Mean ± SE	*N*	Mean ± SE
Baseline	136	3.16 ± 0.17	139	3.2 ± 0.17
12 months	65	2.01 ± 0.27	139	1.9 ± 0.21
24 months	43	1.92 ± 0.46	139	1.7 ± 0.45
36 months	40	1.45 ± 0.28	139	1.5 ± 0.27
Baseline—36 months	39	−2.13 ± 0.35 *P* < .0001	139	−1.68 ± 0.31 *P* < .0001

**Table 3:** 50% Reduction in leaks per day based on diary entries.

	Directly Evaluable	Intent-to-Treat*
12 months	54% (34/63)	55% (74/135)
24 months	64% (27/42)	64% (86/135)
36 months	62% (24/39)	60% (81/135)

*Note: 4 patients had 0 leaks at baseline, thus could not be evaluated for percent change from baseline.

**Table 4:** Incontinence qualify of life questionnaire results.

Visit	Directly Evaluable		Intent-to-Treat	
	*N*	Mean ± SE	*N*	Mean ± SE
Baseline	139	51.2 ± 1.7	139	51.2 ± 1.7
3 months	119	63.9 ± 2.2	139	63.4 ± 2.1
6 months	107	66.4 ± 2.5	139	65.0 ± 2.4
12 months	75	67.7 ± 3.0	139	65.3 ± 2.7
18 months	61	70.2 ± 3.3	139	68.3 ± 3.1
24 months	45	69.0 ± 4.0	139	65.8 ± 3.3
36 months	76	73.0 ± 3.0	139	67.7 ± 4.2
Baseline–36 months	76	21.0 ± 3.1 *P* < .0001	139	16.5 ± 4.3 *P* = .0004

**Table 5:** Improvement in incontinence qualify of life questionnaire scores

	>10-point improvement		Any improvement	
Visit	Directly Evaluable	Intent-to-Treat	Directly Evaluable	Intent-to-Treat
3 months	55% (65/119)	57% (79/139)	69% (82/119)	68% (95/139)
6 months	57% (61/107)	56% (78/139)	73% (78/107)	69% (96/139)
12 months	65% (49/75)	56% (78/139)	80% (60/75)	71% (99/139)
18 months	70% (43/61)	62% (81/139)	82% (50/61)	76% (105/139)
24 months	69% (31/45)	58% (81/139)	82% (37/45)	72% (100/139)
36 months	68% (52/76)	60% (84/139)	78% (59/76)	71% (99/139)

**Table 6:** Incontinence qualify of life questionnaire ≥10-point Improvement, imputed results by severity at baseline.

Visit	Mild	Moderate	Severe
	(*n* = 50)	(*n* = 65)	(*n* = 24)
3 months	42% (21)	60% (39)	79% (19)
6 months	36% (18)	62% (40)	83% (20)
12 months	34% (17)	63% (41)	83% (20)
18 months	32% (16)	78% (51)	79% (19)
24 months	38% (19)	66% (43)	79% (19)
36 months	36% (18)	69% (45)	88% (21)

**Table 7:** Urogenital distress inventory-6.

Visit	Directly Evaluable		Intent-to-Treat	
	*N*	Mean ± SE	*N*	Mean ± SE
Baseline	139	52.7 ± 1.4	139	52.7 ± 1.4
3 months	118	37.2 ± 1.9	139	37.7 ± 1.8
6 months	108	35.6 ± 2.1	139	36.6 ± 1.9
12 months	75	35.1 ± 2.7	139	36.6 ± 2.5
18 months	60	33.7 ± 3.2	139	35.7 ± 3.1
24 months	45	30.4 ± 3.4	139	35.7 ± 4.0
36 months	75	30.1 ± 2.8	139	33.3 ± 4.8
Baseline—36 months	75	−22.6 ± 3.2 *P* < .0001	139	−19.4 ± 5.0 *P* = .0005

**Table 8:** Pad weight.

Visit	Directly Evaluable		Intent-to-Treat	
	*N*	Mean ± SE	*N*	Mean ± SE
Baseline	139	35.5 ± 3.4	139	35.5 ± 3.4
12 months	67	23.6 ± 5.4	139	21.2 ± 4.3
24 months	42	23.2 ± 7.2	139	29.6 ± 8.0
36 months	40	23.0 ± 6.7	139	23.0 ± 8.6
Baseline—36 months	40	−12.2 ± 8.3 *P* = .15*	139	−12.5 ± 9.1 *P* = .18

*Per *t-*test, signed rank test *P* = .03. Two extreme observations are noted. One shows an increase of 196, and the next largest is 85.3.

**Table 9:** 50% Reduction in pad Weight*.

	Directly Evaluable	Intent-to-Treat
12 months	70% (47/67)	68% (95/139)
24 months	69% (29/42)	62% (86/139)
36 months	63% (25/40)	57% (79/139)

*It should be noted that 72 patients have no 12- to 36-month follow-up data available. These patients also appear to differ significantly from those with follow-up data in baseline characteristics in that they had generally less severe symptoms. It is thus important to focus on the multiple imputation results that include values for all patients, particularly for this outcome.

**Table 10:** “Dry” Pad (<1 g).

	Directly Evaluable	Intent-to-Treat
12 months	39% (26/67)	38% (53/139)
24 months	40% (17/42)	30% (42/139)
36 months	38% (15/40)	35% (49/139)

**Table 11:** Analysis of results at 36 months, using different assumptions about outcomes for patients with missing data.

	I-QOL ↑≥10 pts	Moderate-severe @ baseline I-QOL ↑≥10 pts	Any I-QOL increase	50% reduction in pad Weight	“Dry” pad (<1 g)	50% reduction in leaks per day
Women with data available at 3 years	68% (52/76)	75% (36/48)	78% (59/76)	63% (25/40)	38% (15/40)	62% (24/39)

Imputation: all surgeries are failures *and* all LTF are failures	37% (52/139)	40% (36/89)	42% (58/139)	18% (25/139)	11% (15/139)	18% (24/135)

Imputation: all surgeries are failures *and* all LTF are successes	68% (95/139)	71% (63/89)	73% (101/139)	75% (104/139)	68% (94/139)	75% (101/135)

Multiple imputation: all surgeries are failures, with last observation carried forward; multiple imputations applied to lost-to-followup patients	56% (78/139)	69% (61/139)	65% (90/139)	47% (65/139)	25% (35/139)	42% (57/135)

## Abbreviations

IRB: Institutional review board
I-QOL: Incontinence quality of life
SUI: Stress urinary incontinence
QOL: Quality of life
UDI-6: Urogenital distress inventory.
